# Virtual reality vs. tablet for procedural comfort using an identical game in children undergoing venipuncture: a randomized clinical trial

**DOI:** 10.3389/fped.2024.1378459

**Published:** 2024-05-13

**Authors:** Christina Zavlanou, Valentine Savary, Stephanie Mermet, David Sander, Corrado Corradi-Dell’Acqua, David Rudrauf, Yvain Tisserand, Cyril Sahyoun

**Affiliations:** ^1^ Swiss Center for Affective Sciences, University of Geneva, Geneva, Switzerland; ^2^ Division of Pediatric Emergency Medicine, Children’s Hospital of Geneva, Geneva University Hospitals, Geneva, Switzerland

**Keywords:** virtual reality, tablet, pediatrics, venipuncture, pain, anxiety, distraction analgesia, procedural comfort

## Abstract

**Introduction:**

Recent research has explored the effectiveness of interactive virtual experiences in managing pain and anxiety in children during routine medical procedures, compared to conventional care methods. However, the influence of the specific technology used as an interface, 3-dimensions (D) immersive virtual reality (VR) vs. 2D touch screens, during pediatric venipuncture, remains unexamined. This study aimed to determine if immersive VR is more effective than a tablet in reducing pain and anxiety during short procedures.

**Methods:**

An interactive game was designed by clinicians and psychologists, expert in pain theory, hypnosis, and procedural pain and anxiety relief, and was tailored for both VR and tablet use. Fifty patients were randomly assigned to either the Tablet or VR group. The primary outcome measures were pain and anxiety levels during the procedure. Secondary outcome measures included the need for physical restraint, duration of the procedure, enjoyment levels, and satisfaction ratings from both parents and nurses.

**Results:**

Participants, in both groups, had low levels of pain and anxiety. Physical restraint was infrequently used, procedures were brief, and high satisfaction levels were reported by patients, parents, and nurses.

**Discussion:**

This study suggests that the type of technology used as a support for the game has a minimal effect on the child's experience, with both groups reporting low pain and anxiety levels, minimal physical restraint, and high enjoyment. Despite immersive VR's technological advancements, this study underscores the value of traditional tablets with well-designed interactive games in enhancing children's wellbeing during medical procedures.

**Clinical Trial Registration:**

[ClinicalTrials.gov], identifier [NCT05065307].

## Introduction

There is a broad consensus that medical routine procedures are not a pleasant experience. Blood sampling, one of the most common routine procedures, is associated with increased levels of anxiety and pain, especially when the patients undergoing venipunctures are children (1–3). This can lead to both immediate consequences, such as impeding timely diagnosis, and long-term effects, such as causing or intensifying medical avoidance due to traumatic experiences (4, 5).

In the pursuit of effective methods to manage pain and anxiety in young patients, and in consideration for the *pro re nata* approach in using pharmacological pain relief (6), various non-pharmacologic analgesic (NPA) approaches have been conceived. These range from breathing exercises and the use of kaleidoscopes, to distraction cards and music, to hypnosis, acupressure, and utilizing the presence of domestic animals during the procedures (7–9).

Technological advances have prompted an evolution in NPA strategies, with technological-based approaches ranging from displaying existing content such as animated cartoons (10), to developing dedicated solutions such as breathing games using custom-made apparatus (11). Recently, 3-dimension (D) immersive Virtual Reality (VR) has gained significant traction in medical settings, particularly in pediatric units and has been shown to significantly improve the management of pain and anxiety during medical procedures when compared to standard of care (SOC) (12–15).

To the best of our knowledge, no study has tested the effect of using 3D immersive VR vs. more traditional devices such as 2D touch screen tablets to run the interactive game during pediatric venipuncture.

Here, we investigated whether VR could outperform a tablet using the same interactive game, which we specifically designed for brief procedures and to be played in an identical way in both conditions.

Unlike studies that tested similar effects using experimental pain induction and commercial games (16), in this study the comparison was conducted in an ecologically valid context, that is during venous blood sampling in school-aged children, as part of a SARS-CoV-2 seroprevalence study (17).

## Materials and methods

### Study design

This study was a randomized, controlled, parallel-design trial of two non-invasive interventions with between-groups comparison. This publication follows the CONSORT guidelines for reporting randomized controlled trials (18). The study was registered with ClinicalTrials.gov (NCT05065307).

### Study participants

Participants were first-time volunteers participating in a SARS-CoV-2 prevalence study involving venipuncture, which was proposed to randomly contacted families from the area. Enrolled participants were approached to participate concomitantly in our trial, which took place between June and July 2021. The investigators remained unaware of the participants’ identities until they arrived at the site on the day of the procedure.

### Inclusion criteria

• Children and pre-adolescents aged 5–12 years of age.
• Able to understand the French language.

### Exclusion criteria

• Pre-existing photosensitive epilepsy.
• Moderate or severe intellectual disability.
• Parental preference against their child’s use of video screens.
• Comprehension barrier hindering an adequate understanding of the study procedures.
• Physical factors preventing placement of the VR headset (e.g.,: wound on head or face).

### Interventions

#### Interactive game development and age-range selection

The interactive game used in both conditions was developed using a modified Delphi process (19) by a multidisciplinary team, comprised of clinicians specializing in procedural sedation, analgesia, and clinical hypnosis, psychologists specializing in the theory of pain, and VR research developers. The game was developed both for VR and Android tablets (Android Open-Source Project) using Unity (Unity Technologies, San Francisco, USA).

The game’s narration specifically integrated several properties of hypnosis induction—including relaxation and regulation—with a gameplay designed to induce distraction and empowerment.

**Figure 1:**
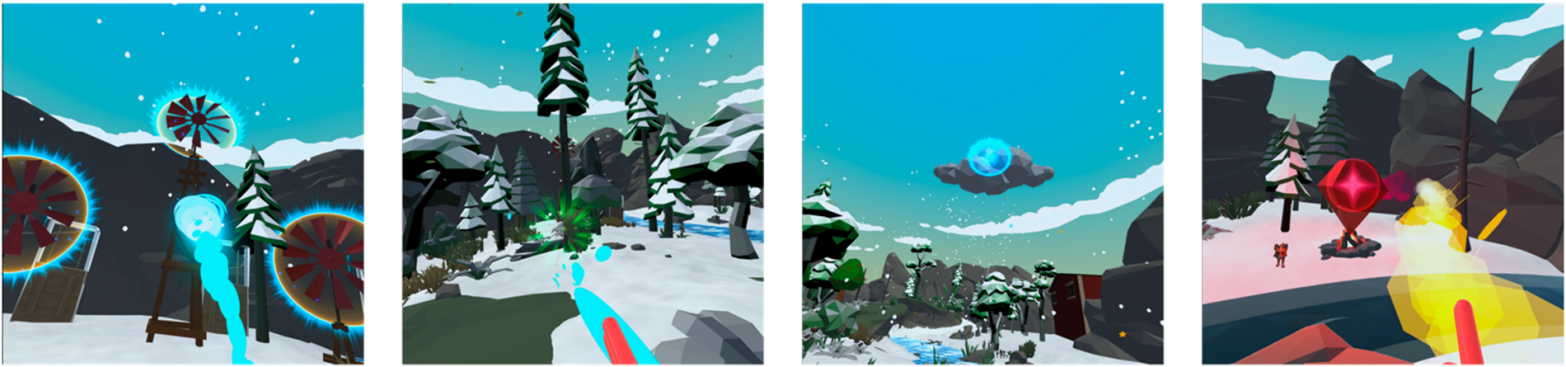
Screen captures of the interactive game missions played by the study participants. From left to right: First mission where the player projects wind to make windmills spin (practice and tourniquet placement phase). Second mission where the player pours water to make plants grow into trees (skin disinfection phase). Third mission where the player throws snowballs onto clouds to make them stop from making snow (venipuncture phase). Fourth mission where the player throws fireballs to light up firepits (repeat venipuncture phase, if necessary). Not displayed: Introductory phase of discovery and breathing exercises, and final phase of reward, where the player is invited to throw fireworks.

The blood sampling schedule was synchronized with the four distinct “missions” of the game, aligning each step of the sampling process—from applying the tourniquet to placing the final bandage—with a corresponding mission (Figure 1).

Venipuncture was chosen as it is one of the most common medical procedures performed in children and remains a common source of unrelieved pain and anxiety. The game was designed for young children (from age 5), as this age group often quickly adapts to computer games and virtual environments, but at the same time has not reached the susceptibility peak of hypnosis which occurs around pre-adolescence (20).

Although some children younger than five can be immersed in VR, we decided not to enroll participants under that cut-off to strengthen confidence in the validity of subjective ratings, which rely on the development around age five of the concept of time, declarative memory, the proficient understanding of averaging, and executive functions such as attentional regulation (21).

#### Data collection procedures

The trial was conducted in two sites, where the SARS-CoV-2 prevalence study took place. One of the sites was located within the confines of the university hospital, and the other in an external office building; both sites featured typical medical consultation rooms.

Participants accompanied by their parents were approached in a waiting area, where the informed consent was filled, as well as a questionnaire, which included information about the children's age, gender, known medical conditions, placement of a Eutectic Mixture of Local Anesthetic (EMLA) patch on the site of the venipuncture, and previous experience with hypnosis and the technologies used for the study.

**Figure 2:**
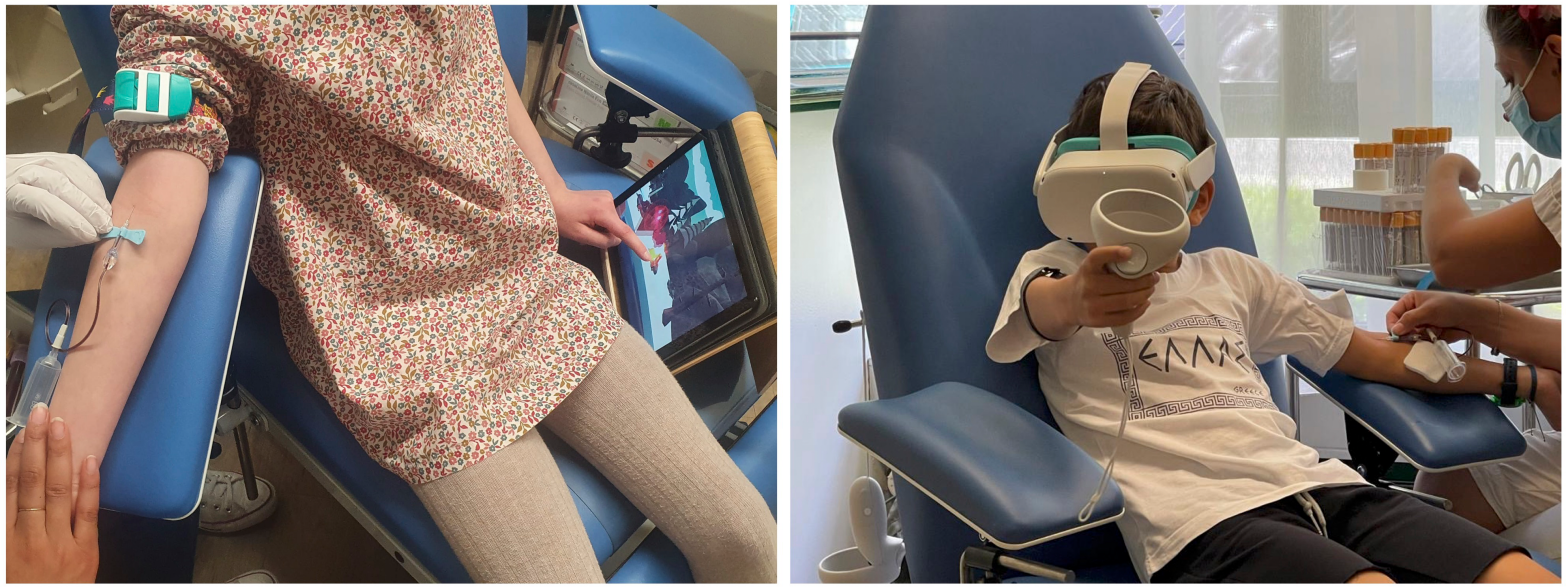
Participants of the tablet group (left) and the VR group (right) during the venipuncture.

Participants were randomly allocated to one of two experimental groups: the “VR” group that played the interactive game using a head-mounted display (Meta Quest 2, Meta, California, USA) and the “Tablet” group that played the same game using a tablet (Lenovo Tab P11, Lenovo, Hong Kong) (Figure 2).

A trained psychologist then filled a graded-graphic rating scale (GRS) questionnaire with the parents and their child. To facilitate understanding, the child was presented with a visual scale to manipulate and asked to point at a number (from 0 to 10) representing their response. The questionnaire investigated:

• Child's self-reported current state anxiety.
• Child's assessment of the potential unpleasantness of the venipuncture.
• The trait anxiety of the child, as related to previous experiences of intravenous procedures or vaccine administration at the pediatrician's office.
• The corresponding trait anxiety that the parents themselves experienced as children. Care was taken to collect parents’ answers in private, to avoid potentiating their child’s anxiety.

The child was then assisted onto the procedure chair, where the gameplay and the interaction with the medium were explained. During that period, a modified Yale Preoperative Anxiety Scale (mYPAS), measuring participant’s hetero-reported current state of anxiety (22, 23) was filled by the psychologist.

The nurse performing the procedure then explained to the child, in a developmentally appropriate manner, how the venipuncture would be carried out. After the arm of the child was visually inspected, a site for the venipuncture was chosen and the game and procedure started.

The second investigator stayed close to the child to ensure comprehension of the game's tasks and the proper course of the procedure. The first game mission aimed at familiarizing the child with the use of the device to interact with the virtual environment and preparing for the procedure through a short breathing exercise. The second game mission corresponded to the period of venipuncture site disinfection. When the third game mission was started, the investigator signaled to the study nurse that it was time to penetrate the skin with the needle. A fourth game mission was designed to accommodate the potential need for a repeat venipuncture, if required.

The child continued playing until a bandage was placed over the puncture site. The second investigator then informed the child that the procedure was finished and that the headset could be removed, or the tablet handed back to the staff, once the game was over.

Following the procedure, the medical staff was asked to rate the difficulty of the venipuncture using a modified version of C-DIVA, rating the visibility and palpability of the vein [mC-DIVA (24)], the number of puncture attempts, the number of persons required to physically restrain the child if needed, the staff's assessment of the pain experienced by the child, whether the game facilitated the procedure, and the staff's overall satisfaction regarding the device's usefulness for the child's wellbeing during the procedure.

Finally, the children and their parents were taken to a private waiting room, where the post-procedure questionnaires were completed (Supplementary Material).

### Outcome measures

#### Primary measures

Two primary outcome measures were assessed in the present study: pain and anxiety.

##### Pain

There are multiple scales available to assess pain in children (25–29). Most however only measure maximum pain felt at a specific moment in time. Given the multidimensional nature of pain, and similar to recently published studies, pain was assessed using three different measures: the maximum pain felt, the unpleasantness associated with the procedure, and the time spent thinking about pain (13, 14, 30–33). To measure pain, we used GRS that included words to describe the lower, upper, and middle bounds of our scale (Supplementary Material). For the outcomes “maximum pain felt” and “unpleasantness associated with the procedure”, we supplemented the GRS with the Faces Pain Scale, a validated self-report measure of pain intensity developed for children 5 years of age and older (34, 35).

##### Anxiety

Similarly, the state of pre-procedure anxiety was measured using a GRS under two different components:

• Self-assessment of the anxiety of the child.
• Hetero-assessment of the anxiety of the child, by the parents.

#### Secondary measures

In addition, secondary outcome measures were considered, as listed below.

##### Participant characteristics (GRS)

• Game immersion.
• Cybersickness (feeling of nausea or vertigo).
• Experienced fun.

##### Procedure characteristics

• Number of attempts necessary to obtain blood sample.
• Number of persons necessary to physically contain the child during the venipuncture.
• Duration of the procedure, from placement of the tourniquet to placement of the final bandage.

##### Parental characteristics (GRS)

• Parental anxiety during the procedure.
• Parental rating of satisfaction.

##### Medical staff characteristics

• Nurse satisfaction.
• Nurse report of usefulness of technology.

### Power analysis

The sample size was set using an effect size obtained from an earlier unpublished pilot study by the authors in which 38 children underwent a similar experimental manipulation and which revealed a Cohen's d = 1, for the mean difference in maximum pain felt. We therefore expected a similar effect size for our five measures in the present study. An a-priori power analysis was conducted for an independent samples t-test, with a significance level of *α* = 0.01 (corresponding to a Bonferroni-corrected *α* for five independent measures), a power level of *β* = 0.80, and an effect size of Cohen's d = 1. The analysis revealed that a total sample size of 48 participants (24 for each group) would detect such an effect. In addition, we increased our target sample size to 60 participants (30 per group), as a safeguard against potential dropout or incomplete data (∼20% increase). Once data collection was completed, the final sample size was 50 participants (25 per group).

### Randomization

Sequence generation: the randomized sequence was created using an online tool by Sealed Envelope Ltd (36).

Allocation concealment mechanism: the randomization sequence was concealed from the study investigators, until informed consent was obtained from the participants and their family.

Implementation: The randomized sequence was generated by investigator CS. Depending on the day of recruitment, participants were enrolled by VS, CZ, or SM, who also assigned participants to an intervention, based on the randomized sequence.

### Blinding

Given the easily recognizable nature of each of the two interventions, no blinding to the interventions could be performed. Blinding of the data analysis was however achieved by engaging an independent analyst (in acknowledgments below) (37).

### Data analysis

A comprehensive inspection showed that the data collected was not normally distributed yet respected equality of variance. To test the differences between the two experimental groups, a non-parametric Mann–Whitney two-tailed test was performed on each main variable. The statistical analysis was conducted with RStudio (2022.07.1, Build 554).

## Results

**Figure 3:**
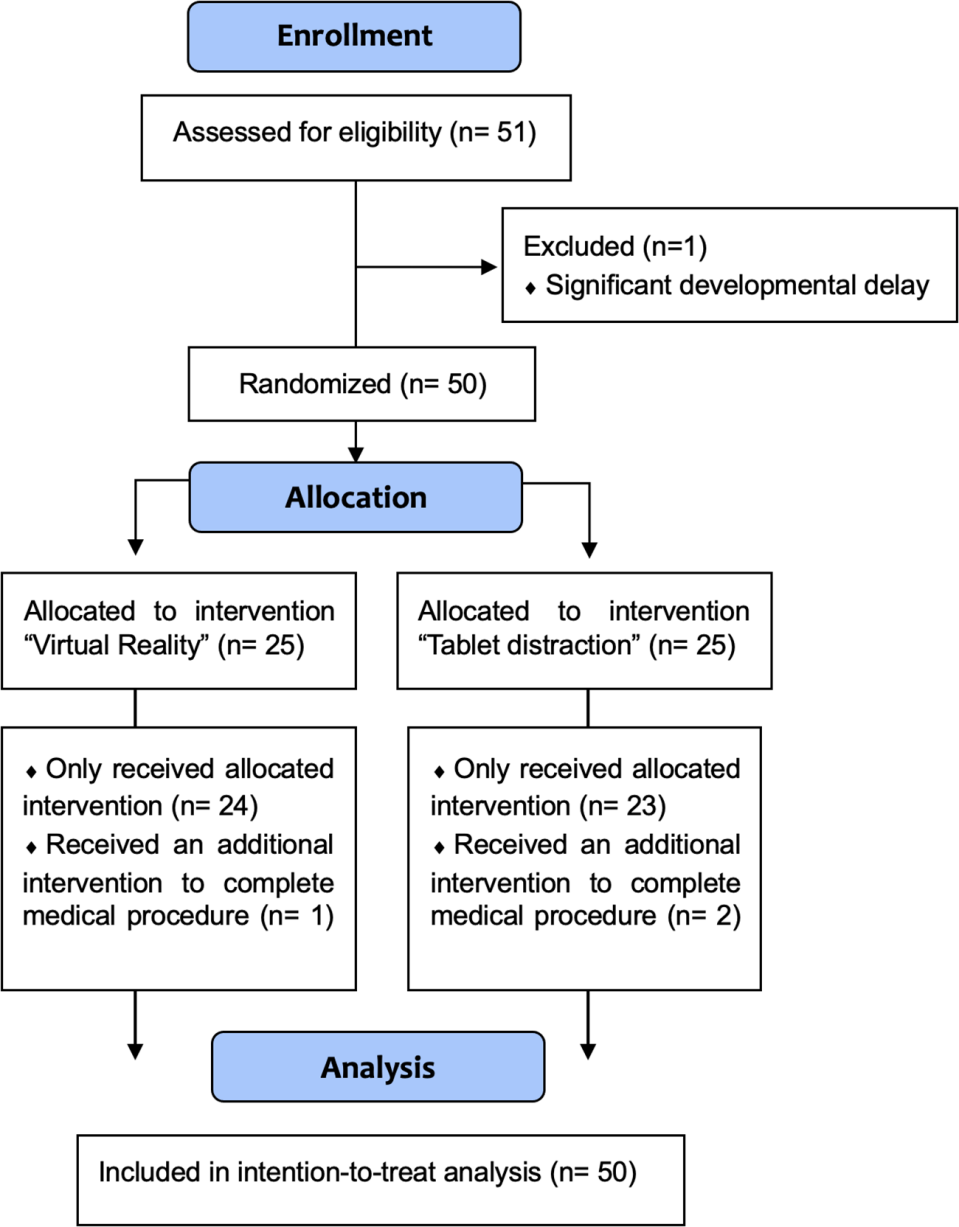
CONSORT flow diagram of participants.

Between June 21 and July 6, 2021, 51 children and their parents were approached for enrolment. Fifty-one consented and one was excluded because of significant developmental delay, which would interfere with the proper use of the VR headset and the tablet device. Participants were randomized to the VR or the tablet group, and all consenting participants (48% females, 52% males) were analyzed in an intention-to-treat analysis (Figure 3). The median age of participants was 8.3 years (IQR = 7.2–10.2) in the Tablet group and 9 years (IQR = 6.3–10.8) in the VR group. There were no significant statistical differences in baseline demographics and characteristics between the two groups (Table 1).

**Table 1:** Baseline participant characteristics of the 50 participants allocated to tablet or VR.

	Tablet (*n* = 25)	VR (*n* = 25)	*p*-value
Age, median (IQR)	8.3 (IQR = 7.2–10.2)	9 (IQR = 6.3–10.8)	0.9615
Gender, *n* (%)			
Female	10 (40%)	14 (56%)	0.395
Male	15 (60%)	11 (46%)	
Child anxiety pre-procedure (GRS 0–10), mean (SD)	4.00 (3.22)	3.16 (2.97)	0.342
Child anticipated procedure unpleasantness (GRS 0–10), mean (SD)	4.04 (2.74)	3.57 (3.16)	0.584
Needle fear at routine medical visit (evaluation by parent) (GRS 0–10), mean (SD)	3.40 (3.32)	3.77 (3.34)	0.703
Parent anxiety pre-procedure (GRS 0–10), mean (SD)	0.65 (1.3)	1.45 (2.04)	0.142
mYPAS score (23.33–100), mean (SD)	41.8 (10.2)	38.7 (11.4)	0.31
mC-DIVA score (0–4, 4 being most difficult), mean (SD)	0.56 (1.23)	0.40 (1.00)	0.836
EMLA patch use, *n* (%)	20 (80%)	24 (96%)	0.192
prior experience with VR, *n* (%)	12 (48%)	10 (40%)	0.776
prior experience with Tablet, *n* (%)	23 (92%)	21 (84%)	0.663
prior experience with Hypnosis, *n* (%)	2 (8%)	1 (4%)	1

VR, virtual reality; IQR, interquartile range; SD, standard deviation; GRS, graphic rating scale; mYPAS, modified Yale preoperative anxiety scale; mC-DIVA, modified comprehensive difficult intravenous access; EMLA, eutectic mixture of local anesthetics.

### Primary outcome analysis

**Figure 4:**
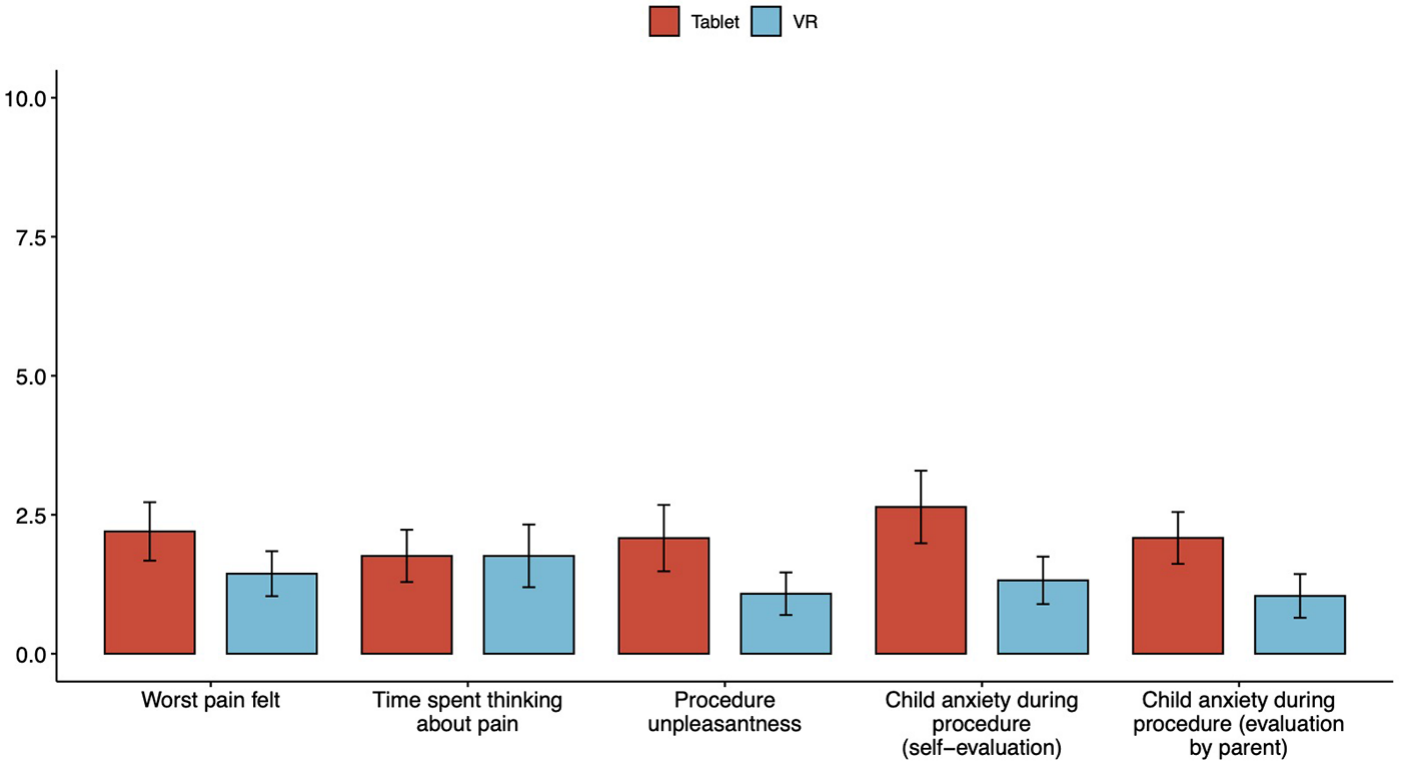
Analysis of primary outcome measures, showing mean and standard error.

Mean differences in worst pain felt (2.2 (SD = 2.63) vs. 1.44 (SD 2.02), *p* = 0.284), time spent thinking about pain (1.76 (SD = 2.35) vs. 1.76 (SD = 2.82), *p* = 0.385), procedure unpleasantness (2.08 (SD = 2.99) vs. 1.08 (SD = 1.91), *p* = 0.417), self-assessed child anxiety during procedure (2.64 (SD = 3.26) vs. 1.32 (SD = 2.14), *p* = 0.208) and parental hetero-assessment of child anxiety during the procedure (2.08 (SD = 2.28) vs. 1.04 (SD = 1.97), *p* = 0.06) were not statistically significant between the Tablet and the VR groups, respectively (Table 2, Figure 4).

**Table 2:** Summary statistics and two-tailed Mann–Whitney results of the main outcome measures.

Treatment conditions	Tablet			VR				
Variables	*N*	Mean	SD	*N*	Mean	SD	Test	*p*-value
Worst pain felt	25	2.2	2.63	25	1.44	2.02	W = 365	0.284
Time spent thinking about pain	25	1.76	2.35	25	1.76	2.82	W = 355.5	0.385
Procedure unpleasantness	25	2.08	2.99	25	1.08	1.91	W = 350.5	0.417
Child anxiety during procedure (self-evaluation)	25	2.64	3.26	25	1.32	2.14	W = 373.5	0.208
Child anxiety during procedure (evaluation by parent)	24	2.083	2.28	25	1.04	1.97	W = 388.5	0.06

Statistical significance markers: * *p*<0.01; ** *p*<0.05; *** *p*<0.01

### Secondary outcome analysis

Altogether, participants in the VR group did not complain about motion sickness and reported feeling highly immersed in the virtual environment (8.44/10, SD = 2.60). In both groups, children reported elevated levels of “fun” (8.36/10 (SD = 2.55) in tablet group and 8.96/10 (SD = 1.84) in VR group). The percentage of subjects requiring physical restraint was low (8% in Tablet vs. 16% in VR) and the number of venipuncture attempts were similar (88% for one attempt in tablet vs. 92% in VR) in both groups. Nurses and parents were highly and similarly satisfied with both hardware solutions (*>*9/10 in all rating groups and for both conditions). The mean duration of the procedure, from placement of the tourniquet to placement of the final bandage was 155 s (SD = 46) in Tablet vs. 168 s (SD = 78) in VR. None of the secondary outcome analyses showed any significant difference between tablet and VR. Results are reported in Table 3.

**Table 3:** Analysis of secondary outcome measures.

Treatment conditions	Tablet	VR
Variables		
Physical restraint, *n* (%)		
0	23 (92%)	21 (84%)
1 person	1 (4%)	4 (16%)
2 persons	1 (4%)	0 (0%)
Venipuncture attempts, *n* (%)		
1 attempt	22 (88%)	23 (92%)
2 attempts	3 (12%)	2 (8%)
Nausea, *n* (%)	0 (0%)	0 (0%)
Vertigo, *n* (%)	0	0
Duration of the procedure, seconds (SD)	155 (46)	168 (78)
Intervention usefulness evaluated by nurse, GRS (0–10), mean (SD)	9.35 (1.5)	9.6 (1.2)
Nurse satisfaction, GRS (0–10), mean (SD)	9.52 (2.1)	9.24 (1.7)
Parent satisfaction, GRS (0–10), mean (SD)	9.25 (1.3)	9.76 (0.66)
Parent anxiety during procedure, GRS (0–10), mean (SD)	0.92 (1.64)	1.08 (2.04)
Game immersion, GRS (0–10), mean (SD)	5.84 (3.83)	8.44 (2.6)
Fun experienced, GRS (0–10), mean (SD)	8.36 (2.55)	8.96 (1.84)
Additional intervention to complete procedure, *n* (%)		
More VR	1 (4%)	1 (4%)
Parents distracted the child	1 (4%)	0 (0%)

SD, standard deviation; GRS, graphic rating scale; VR, virtual reality.

### Exploratory analysis

Relationships between selected hypothesized predictors (age, gender, anxiety pre-procedure, mYPAS pre-procedure, anticipated pre-procedure unpleasantness, physical restraint, mC-DIVA, EMLA use, game immersion, fun) and the primary outcome measured revealed that within the Tablet group only, the greater the expected unpleasantness of the procedure, the greater the unpleasantness felt during the procedure (*p* = 0.028), and the greater the anxiety of the child before the procedure, the greater the anxiety felt during the procedure (*p* = 0.003). These effects were not found within the VR group. The results are presented in Tables 4, 5.

**Table 4:** Summary table of the resampled *p*-value obtained during exploratory analyses of the effects of selected variables on the primary outcome measures for the treatment group “tablet” (ANOVA table with resampling test using freedman-lane to handle nuisance variables with 500 permutations).

		Primary outcome measures				
		Time spent thinking about pain	Procedure unpleasantness	Worst pain felt	Child anxiety during procedure (self-evaluation)	Child anxiety during procedure (evaluation by parent)
Selected variables	Age	0.430	0.866	0.715	0.206	0.914
	Child anxiety pre-procedure (self-evaluation)	0.625	0.898	0.484	0.003**	0.205
	Expected unpleasantness of the procedure (self-evaluation)	0.887	0.028**	0.961	0.892	0.385
	Physical restraint	0.169	0.297	0.264	0.666	0.129
	mYPAS score	0.477	0.320	0.880	0.762	0.567
	mC-DIVA score	0.737	0.631	0.929	0.303	0.580
	Game immersion	0.424	0.159	0.466	0.467	0.833
	Fun experienced	0.793	0.776	0.988	0.793	0.652

mYPAS, modified Yale preoperative anxiety scale; mC-DIVA, modified comprehensive difficult intravenous access.

Statistical significance markers: *p < 0.1; **p < 0.05; ***p < 0.01.

**Table 5:** Summary table of the resampled *p*-value obtained during exploratory analyses of the effects of selected variables on the primary outcome measures for the treatment group “VR” (ANOVA table with resampling test using freedman-lane to handle nuisance variables with 500 permutations).

		Primary outcome measures				
		Time spent thinking about pain	Procedure unpleasantness	Worst pain felt	Child anxiety during procedure (self-evaluation)	Child anxiety during procedure (evaluation by parent)
Selected variables	Age	0.923	0.406	0.100	0.951	0.376
	Child anxiety pre-procedure (self-evaluation)	0.574	0.430	0.874	0.892	0.388
	Expected unpleasantness of the procedure (self-evaluation)	0.962	0.380	0.362	0.680	0.203
	Physical restraint	0.447	0.102	0.185	0.246	0.353
	mYPAS score	0.410	0.959	0.923	0.434	0.063
	mC-DIVA score	0.390	0.010**	0.001***	0.010**	0.001***
	Game immersion	0.537	0.620	0.721	0.596	0.067
	Fun experienced	0.106	0.164	0.022	0.626	0.124

mYPAS, modified Yale preoperative anxiety scale; mC-DIVA, modified comprehensive difficult intravenous access; VR, virtual reality.

Statistical significance markers: *p < 0.1; **p < 0.05; ***p < 0.01.

## Discussion

In this randomized controlled trial, children undergoing venipuncture using an interactive game specifically designed for such procedures showed low levels of pain and anxiety, high level of experienced fun and low incidence of physical restraint, irrespective of the technology that was used. The procedure duration was equally short in both experimental conditions, parents/caregivers showed low levels of anxiety, and both caregivers and medical staff reported elevated levels of satisfaction.

### Effect of the technology

Multiple studies have studied the interest of VR as compared to SOC for venipuncture (38, 39), showing variable effect of VR on pain, anxiety, reduction of fear and physical restraint. In several of these studies, VR appeared superior to SOC (40–42). In our pilot study, conducted in a more stressful environment and from which the sample size used in this study was derived, VR also appeared superior to SOC.

However, no study had looked at the effect of the type of technology used as an alternative to SOC, e.g., immersive VR vs. tablets, based on identical interactive games or virtual environments, on a child's procedural comfort during venipuncture. Ryu et al. (43) compared an identical three-minute educational intervention in VR vs. tablet to help children better tolerate chest radiography. Their results showed that pre-procedural education using VR significantly reduced anxiety and distress in children and improved the efficiency of the procedure by reducing the overall duration of the procedure and the need for repeated takes, as compared to the tablet. Venipuncture, a procedure usually associated with elevated levels of anxiety, is quite different from chest radiography. Our initial hypothesis was that the immersive nature of VR would improve the overall clinical experience. However our results, particularly the small number of children requiring restraint, as compared to other cohorts and as discussed below, suggest that what may matter most is the actual design of the interactive game, which in our study was designed and implemented to specifically follow the different steps of the venipuncture procedure, and mimic the support of the child by a dedicated person during the venipuncture, as in the work of Child Life Specialists (44), which was not available in our healthcare setting. Further studies should be pursued to test this latter hypothesis.

### Procedure duration

Procedure duration, from the time of tourniquet placement to final bandage, was short in both conditions (2.6 min for tablet vs. 2.8 min in VR). Although the amount of blood which needed to be collected was small, pediatric clinicians will appreciate the particularly short duration of the procedure, even though studies specifically assessing standard venipuncture duration in children are lacking. In one adult study, mean phlebotomy time, timed from the moment of phlebotomist initial hand hygiene to the moment of final hand hygiene, was measured to be 4–4.5 min, depending on the experience of the person doing the procedure (45). Our study, although measuring procedure duration differently, reveals an even shorter time, even though pediatric venipuncture is generally considered more difficult than the procedure in adult. Indeed, the nurses participating in our study noted that once the study participants started to play, they appeared so focused and immersed in the tasks at hand that it was rare for them to require reassurance, allowing the nurses to proceed swiftly with the venipuncture. This short procedure duration, while concomitantly focusing on creating a positive experience for the child, may prove to be useful in fast-paced clinical environments where efficiency is essential.

### Physical restraint

One overarching goal for clinicians, when tackling procedural comfort in pediatrics, is to help children return to subsequent medical care feeling trust towards the healthcare system. Beyond pain and anxiety, physical restraint increases the risk of emotional trauma, loss of trust, and subsequent post-traumatic stress disorder, and can be regarded as a proxy for quality of care. In a study by Chan et al. comparing VR to standard of care (42), the authors found that VR significantly reduced the need for restraint during pediatric venipuncture in the outpatient laboratory (20% vs. 67% restraint by more than one person). In our study, physical restraint was rarely needed under both conditions, with 4% of the children in the VR group and none in the tablet group having to be restrained by more than one person, contributing to further building trust during medical procedures.

### Expected unpleasantness and pre-procedure anxiety

The exploratory analysis revealed differences between the VR and the Tablet conditions. In the Tablet group only, child pre-procedure anxiety appears to significantly correlate with self-reported child anxiety during the procedure and expected procedure unpleasantness with actual procedure unpleasantness. This difference between the two conditions may suggest that VR is more effective at immersing children into the game than a tablet device, perhaps pointing to the advantage of such technology. In clinical practice and when using a Tablet for procedural comfort, this correlation could perhaps be reduced by starting the interactive game well in advance, for example in the waiting area of a medical clinic, similarly to results reported on the management of preoperative anxiety using cognitive behavioral therapy, guided imagery relaxation and hypnosis (46).

### Trade-off in game design between the study devices

Aiming for the creation of an identical game in both the tablet and VR groups did involve some design trade-offs. For example, as haptic feedback on tablets differs considerably from that achieved using the VR controllers, we decided not to include it in either case, although it is hypothesized that this could have enhanced the experience.

Moreover, due to the nature of the venipuncture procedure, the virtual environment of each game's mission, in the tablet group, was static. Indeed, the three-axial rotation of the tablet could have been employed to create the impression of being placed within a 360° space, however, this was not feasible, as the subjects could not use both hands to rotate the tablet, while interacting with the game. Thus, the study participants could locate the virtual targets on the screen without having to search for them, unlike the participants playing the game in VR, which may have rendered the tablet game less explorative and challenging than in VR.

An additional element opted for, given the constraints imposed by the disparity between the two platforms, is the static nature of the experience. That is, we could have provided a gameplay on tablets in which the user could move around in the virtual environment, as in a “platform game”. This was purposely avoided as in VR, aside from the obvious need for some immobility during venipuncture, moving around the environment could have lead to motion sickness.

We suggest that future game development targeting only one of the two devices consider these elements as ways to improve the overall user experience.

### Limitations

The most important limitation of our study is that children who came for venipuncture did so mostly on a voluntary basis, as part of a COVID-19 serological study, with middle to low baseline mYPAS scores, in children who for the majority, had EMLA patches in place. Our study may have shown different results if performed in more acute settings such as emergency departments or at the laboratory where children and their parents tend to be more vulnerable and fearful, and hence could perhaps be more affected by the virtual environment.

Another potential limitation may be choice of pain and anxiety measures used in this study. While multiple observational and self-reported measures exist to document pain and anxiety in children, to date, there is no perfect pediatric measure nor a reliable vital sign (47–49). We do however believe that the measures we used, as tested in other studies described above, do offer an accurate multimodal description of the experience lived by the subjects.

Another potential limitation is the use of an additional person who stayed close to the child to ensure comprehension of the game's tasks and the proper course of the procedure, throughout the venipuncture. This may appear unfeasible in medical settings where staffing is scarce, however, *in situ* and outside the purpose of this study, the person filling this role is usually a nurse’s aide who is already involved in the patient's procedure and who is helping hold the arm of the child in the appropriate position, such as is common practice in pediatrics.

## Conclusion

In conclusion, according to this randomized controlled trial of children undergoing venipuncture, the technology used as an interface for the interactive game appears to have only a minor impact on the child's experience. In both conditions, children experienced low levels of pain, of anxiety and of physical restraint, and elevated levels of fun, and parents and nurses were highly satisfied. Despite the advent of newer technology such as VR, this study highlights the value of traditional tablet devices, and perhaps other forms of non-technological distraction methods, when well-designed interactive experiences are implemented, for improving procedural comfort.

Future research is needed to further understand what makes the most difference in a child's experience when using such technology in a given clinical context and how generalizable such solutions may be. An environment and gameplay specifically designed for a procedure may prove to be most important.

## Data Availability

The raw data supporting the conclusions of this article will be made available by the authors, without undue reservation.
