# Supplementary material for: Virtual reality vs. tablet for procedural comfort using an identical game in children undergoing venipuncture: a randomized clinical trial
Source: Front Pediatr. 2024 May 13;12:1378459. doi: 10.3389/fped.2024.1378459 (PMC11128582; doi:10.3389/fped.2024.1378459)
Supplement: Supplementary file 1 [file Datasheet1.pdf]

Renard Corona study

Start of Block: Indications de base

0
Questions démographiques

Q93 Site

- ○ Jean-Violette (1)
- ○ Plateforme pédiatrie (2)

Q84 Critères d'inclusion

	Oui (1)	Non (2)
A entre 5 et 12 ans inclus (1)	○	○

Skip To: End of Survey If Critères d'inclusion = Non

Q85 Critères d'exclusion (à vérifier avec les parents)

	Non (1)	Oui (2)
Refus parental d'écrans (2)	○	○
Epilepsie photosensible (interdiction d'écrans) (3)	○	○
Trouble développemental modéré ou sévère (4)	○	○
Plaie ou autre barrière physique du casque (5)	○	○
Barrière de compréhension de la part du parent ou de l'enfant (6)	○	○
L'enfant a vu un membre de sa famille participer à l'étude avant lui (15)	○	○

Skip To: End of Survey If Critères d'exclusion (à vérifier avec les parents) = Oui

Q94
Expliquer l'étude aux parents et à l'enfant puis faire signer consentement

Pourquoi menons-nous ce projet de recherche ?
Nous voulons savoir **à quel point les supports informatiques permettent de diminuer le stress et la douleur de l'enfant pendant une prise de sang**, et de lui faire vivre une expérience mois désagréable. Grâce aux résultats de cette étude, nous serons en mesure de mieux comprendre quel type de support informatique réduit plus la douleur et l'anxiété pendant un tel soin.

Que doit faire la patiente/le patient en cas de participation ?
Si vous acceptez de participer à ce projet, **nous proposerons à votre enfant de jouer à un jeu vidéo sur un des deux supports informatiques choisit au hasard, pendant la prise de sang qui est prévue ce jour. Ce jeu dure environ 7 minutes. Nous mettrons un petit capteur sur le bras de votre enfant pour mesurer ses battements de cœur pendant la prise de sang, et vous poserons ainsi qu'à votre enfant quelques questions avant et après le soin, pour comprendre comment ce soin a été vécu** en terme de confort, de douleur, mais aussi en terme d'amusement.

Quels sont les bénéfices et les risques liés à la participation au projet ?

Votre participation au projet apportera à votre enfant une distraction pendant sa prise de sang. Quant à eux, les résultats du projet pourraient se révéler importants par la suite pour réduire l'anxiété et l'intensité de la douleur ressentie par d'autres enfants pendant une prise de sang ou autre procédure médicale.
C**ette étude pose peu de risques à votre enfant. Très rarement, le jeu vidéo peut provoquer chez certains enfants de légers vertiges ou nausées. Ces symptômes se résolvent généralement dans les heures qui suivent.**

Page Break

*

9 Âge de l'enfant (Nombre d'ANNEES):

____

*

Q78 Âge de l'enfant (Nombre de mois additionnels au nombre d'années, de 1 à 11; Par exemple, mettre "3" pour 5 ans 3 mois):

____

Q95
Selectionnez le support informatique selon tableau de randomisation et en fonction de l'âge de l'enfant

6 Condition expérimentale

- ○ Réalité Virtuelle (1)
- ○ Tablette (2)

Q103 ID enfant randomisé

____

10 Genre de l'enfant:

- ○ M (1)
- ○ F (2)

12 Antécédents comportementaux/développementaux

- ☐ Trouble du Déficit de l'Attention avec/ou sans Hyperactivité (1)
- ☐ Trouble développemental léger (3)
- ☐ Autre (4)
- ☐ Aucun (5)

Display This Question:

If Antécédents comportementaux/développementaux = Autre

Q79 Quel autre antécédent?

____

Q86 EMLA posé ?

- ○ Oui (1)
- ○ Non (2)

Display This Question:

If EMLA posé ? = Oui

13 Depuis combien de temps l'EMLA est posé?

- ◯ Moins de 30 min (6)
- ◯ 30 min (1)
- ◯ 45 min (2)
- ◯ 1 heure (3)
- ◯ Plus d'une heure (4)

Display This Question:

If EMLA posé ? = Oui

Q102 L'EMLA a-t-elle été enlevée avant le soin ?

- ◯ Non (1)
- ◯ Oui depuis 15min (2)
- ◯ Oui depuis 30min (3)
- ◯ Oui depuis plus de 30min (4)

End of Block: Indications de base

Start of Block: Questionnaire pour Enfant (avant le soin)

14
Questions pour Enfant (pré)

Q81
Es tu anxieux ou inquiet, ou as tu peur de faire le soin ? (Si non, selectionner 0).

Si oui, combien ?

	Pas du tout	Un peu	Moyennement	Beaucoup	Extrêmement	Not Applicable

	0	1	2	3	4	5	6	7	8	9	10
Anxiété enfant pre ()											

15
Est ce que tu penses que le soin sera agréable ? (Si oui, selectionner 0).

Si non, combien désagréable ?

	Agréable	Un peu désagréable	Moyennement désagréable	Très désagréable	Extrêmement désagréable	Not Applicable

	0	1	2	3	4	5	6	7	8	9	10
Agréabilité du soin enfant pré ()											

End of Block: Questionnaire pour Enfant (avant le soin)

Start of Block: Questionnaire pour Parents (avant le soin)

16
Questions pour Parent (pré)

17 Combien êtes-vous inquiet.e ou anxieux.se concernant la prise de sang de votre enfant?

	Pas du tout	Un peu	Moyennement	Beaucoup	Extrêmement	Not Applicable
	0 1 2 3 4 5 6 7 8 9 10					
Anxiété du parent pré ()						

End of Block: Questionnaire pour Parents (avant le soin)

Start of Block: Questionnaire pour expérimentateur: mYALE

Q101
Infirmier.ière étudiante présente le support informatique et explique son fonctionnement à l'enfant.

Ne pas encore mettre le casque sur le patient ni lancer le jeu

Q100
mYALE (à remplir par médecin étudiant) basé sur observations AVANT début de la prise de sang

19 Activité

	1: Regarde autour de lui, curieux, peut explorer les objets, ou reste calme (1)	2: N'explore pas, peut regarder vers le bas, peut remuer nerveusement les mains ou sucer son pouce (son doudou) (2)	3: Se tortille, bouge sur la table, peut repousser le masque (3)	4: Tente activement de s'échapper, repousse avec les pieds et les mains, peut bouger tout le corps (4)
Activité (1)	○	○	○	○

20 Vocalisations

	1: Pose des questions, fais des commentaires, babille (1)	2: Répond aux questions des adultes mais à voix basse, "baby-talk", ou ne fait que des signes de tête (2)	3: Silencieux, aucun son, ne répond pas aux adultes (3)	4: Pleurniche, se plaint, gémit (4)	5: Pleure, peut crier "non" (5)	6: Pleure et crie de façon continue (audible continuellement) (6)
Vocalisations (1)	○	○	○	○	○	○

21 Expression émotionnelle

	1: Manifestement heureux (1)	2: Neutre, pas d'expression faciale visible (2)	3: Inquiet (triste) ou effrayé ; yeux effrayés, tristes ou pleins de larmes (3)	4: En détresse, pleure, totalement bouleversé, peut avoir les yeux écarquillés (4)
Expression émotionnelle (1)	○	○	○	○

22 État d'éveil apparent

	1: Alerte, regarde occasionnellement autour de lui, observe ce que le soignant fait (1)	2: Renfermé sur lui même, tranquillement assis, peut sucer son pouce, ou visage tourné vers l'adulte (2)	3: Vigilant, regarde rapidement tout autour de lui, peut sursauter aux bruits, yeux grands ouverts, corps tendu (3)	4: État de panique, pleurniche, peut pleurer ou repousser les autres, se détourne (4)
État d'éveil apparent (1)	○	○	○	○

23 Utilisation du parent

	1: Occupé à jouer, assis tranquillement, ou engagé dans un comportement approprié par rapport à son âge et n'a pas besoin du parent; peut interagir avec le parent si celui-ci initie l'interaction (1)	2: Fait appel au parent (approche le parent silencieux et lui parle), recherche et accepte le réconfort, peut s'appuyer contre le parent (2)	3: Regarde le parent silencieusement, regarde de manière apparente les actions, ne recherche ni le réconfort ni le contact, mais les accepte quand ceux-ci sont proposés ou s'accroche au parent (3)	4: Garde le parent à distance ou peut activement se retirer du parent, peut repousser le parent également ou désespérément s'accrocher à celui-ci et ne pas lâcher prise (4)
Utilisation du parent (1)	○	○	○	○

End of Block: Questionnaire pour expérimentateur: mYALE

Start of Block: Chrono

Page Break

27

Permettez à l'infirmière d'enlever l'EMLA (si posé) et de décider sur quel bras la prise de sang sera faite.
Une fois la décision prise et l'enfant a comprit que le soin va débuter, commencer le jeu sur le support informatique.

Lancer le chronomètre (en cliquant sur la pédale) une fois le garrot posé pour la prise de sang.

Arrêter le chronomètre (en cliquant sur la pédale) une fois le sparadrap ou pansement posé

Page Break

Q98
Permettre à l'enfant de finir le jeu en entier et lancer des feux d'artifices (7 minutes total).
Une fois le jeu finit, l'étudiant accompagnant l'enfant lui dira, calmement:

Casque VR: "Voilà, ton soin est terminé. Quand tu seras prêt, tu pourras revenir vers nous, et toi même enlever ton casque des yeux, merci d'avoir joué"

Tablette: "Voilà, ton soin est terminé, merci d'avoir joué".

End of Block: Chrono

Start of Block: Refus/acceptation

24 Acceptation du support informatique

- Oui (1)
- Non (2)

Display This Question:

If Acceptation du support informatique = Non

25 Si non, pourquoi?

- Plus anxieux avec le support informatique (1)
- Demande à regarder la procédure (2)
- Autre (3)

Skip To: End of Survey If Si non, pourquoi? = Plus anxieux avec le support informatique

Skip To: End of Survey If Si non, pourquoi? = Demande à regarder la procédure

---

Display This Question:

If Si non, pourquoi? = Autre

26 Si autres est sélectionné?

____

Skip To: End of Survey If Condition: Si autres est sélectionné?&... Is Not Empty. Skip To: End of Survey.

End of Block: Refus/acceptation

Start of Block: Questionnaire pour Soignant (post)

46
Questions pour Soignant (post)

---

47 Visibilité des veines après le garrot installé

○ Visibles (1)

○ Pas visibles (2)

---

48 Palpabilité des veines après le garrot installé

○ Palpables (1)

○ Pas palpables (2)

Q87 Succès de la procédure

○ Réussie (1)

○ Echouée (2)

Display This Question:

If Succès de la procédure = Réussie

49 Si réussie:

- ◯ Du premier coup (1)
- ◯ J'ai dû chercher la veine avec le cathéter (2)
- ◯ J'ai du traverser la peau plus d'une fois (3)

Display This Question:

If Succès de la procédure = Echouée

Q88 Si échouée:

- ◯ Enfant trop anxieux/appeuré (1)
- ◯ Enfant a trop mal (4)
- ◯ Pas de retour veineux satisfaisant (2)
- ◯ Autre (3)

Display This Question:

If Si échouée: = Autre

Q89 Si autre raison, laquelle?

____

---

50 Combien de fois avez vous dû traverser la peau?

- 1 (4)
- 2 (5)
- 3 (6)
- 4 (7)
- 5 (8)

51 Combien de personnes ont dû tenir l'enfant pendant la procédure?

- 0 (4)
- 1 mais juste en prévention et sans contention (5)
- 1 (7)
- 2 (8)
- 3 (9)
- 4 (10)

Q90 Combien de douleur pensez-vous que l'enfant a ressenti pendant la procédure médicale?

	Pas de douleur	Douleur faible	Douleur modérée	Douleur forte	Pire douleur imaginable	Not Applicable
	0 1 2 3 4 5 6 7 8 9 10					
Douleur hétérorapportée soignant ()						

56 Combien êtes-vous satisfait de la prise en charge du bien-être de l'enfant aujourd'hui?

	Pas du tout	Un peu	Moyennement	Beaucoup	Extrêmement	Not Applicable
	0 1 2 3 4 5 6 7 8 9 10					
Satisfaction soignant ()						

57 Combien le support informatique vous a-t-il facilité la prise de sang?

	Pas du tout	Un peu	Moyennement	Beaucoup	Extrêmement	Not Applicable
	0 1 2 3 4 5 6 7 8 9 10					
Facilitation du travail soignant ()						

Q92 Combien d'expérience avez vous dans les prises de sang?

	Pas du tout	Un peu	Moyennement	Beaucoup	Extrêmement	Not Applicable
	0 1 2 3 4 5 6 7 8 9 10					
Expérience soignant ()						

52 L'enfant a-t-il eu recu une intervention supplémentaire pour compléter la procédure?

- Oui (1)
- Non (2)

Display This Question:

If L'enfant a-t-il eu recu une intervention supplémentaire pour compléter la procédure? = Oui

53 Si oui, quelle intervention ?

- ◯ Plus de jeu du renard sur réalité virtuelle (1)
- ◯ Plus de jeu du renard sur tablette (2)
- ◯ Téléphone/écran des parents (4)
- ◯ Autre (8)

Display This Question:

If Si oui, quelle intervention ? = Autre

54 Si autre intervention, laquelle ?

____

End of Block: Questionnaire pour Soignant (post)

Start of Block: Questionnaire pour Enfant (Post)

29
Questions pour Enfant (post)

32
As-tu eu mal pendant la procédure ? (Si non, selectionner 0).

Si oui, quel a été le maximum de ta douleur?

	Pas de douleur	Douleur faible	Douleur modérée	Douleur forte	Pire douleur imaginable	Not Applicable

0 1 2 3 4 5 6 7 8 9 10

Douleur senso enfant ()	

30 Combien de temps tu as passé à penser à la douleur pendant la prise de sang?

	Jamais	Un peu de temps (une fois)	la moitié du temps	Longtemps	Tout le temps	Not Applicable

0 1 2 3 4 5 6 7 8 9 10

douleur cognitive enfant ()	

31
Est ce que que le soin a été agréable ? (Si oui, selectionner 0).

Si non, combien désagréable ?

	Agréable	Pas du tout désagréable	Un peu désagréable	Moyennement désagréable	Extrêmement désagréable	Not Applicable

0 1 2 3 4 5 6 7 8 9 10

Douleur affective enfant ()	

Q82
Etais-tu anxieux ou inquiet ou as-tu eu peur pendant que tu recevais ton soin? (Si pas du tout, selectioner 0)

Si oui, combien ?

	Pas du tout	Un peu	Moyennement	Beaucoup	Extrêmement	Not Applicable

0 1 2 3 4 5 6 7 8 9 10

Anxiété post enfant ()	

Q76
Est-ce que tu avais l'impression d'être vraiment en train de jouer dans la nature? (Si non, selectionner 0)

Si oui, combien?

	Pas du tout	Un peu	Moyennement	Beaucoup	Tout le temps	Not Applicable

0 1 2 3 4 5 6 7 8 9 10

Sentiment de présence ()	

34
As-tu ressenti des nausées (envie de vomir) durant le jeu? (Si non, selectionner 0)

Si oui, combien?

	Jamais	Un peu	Souvent	Très souvent	Tout le temps	Not Applicable

	0	1	2	3	4	5	6	7	8	9	10
Nausée ()											

35 As-tu ressenti de vertiges (l'impression de tomber) durant le jeu? (Si non, selectionner 0)

Si oui, combien?

	Jamais	Un peu	Souvent	Très souvent	Tout le temps	Not Applicable

	0	1	2	3	4	5	6	7	8	9	10
Vertige ()											

36
T'es tu amusé durant l'expérience d'aujourd'hui? (Si non, selectionner 0)

Si oui, combien?

	Pas du tout	Un peu	Moyennement	Beaucoup	Énormément	Not Applicable

	0	1	2	3	4	5	6	7	8	9	10
Amusement ()											

Q83 As tu déjà eu une expérience dans le passé avec:

	Non (1)	Oui (2)
Réalité Virtuelle (1)	○	○
Jeux sur tablette (2)	○	○
Hypnose (3)	○	○

End of Block: Questionnaire pour Enfant (Post)

Start of Block: Questionnaire pour Parents (post)

39
Questions pour Parent (post)

44 Selon vous, combien votre enfant a eu mal pendant la prise de sang?

	Pas de douleur	Douleur faible	Douleur modérée	Douleur forte	Pire douleur imaginable	Not Applicable

0 1 2 3 4 5 6 7 8 9 10

Douleur hétérorapportée parent ()

43 Selon vous, combien votre enfant était inquiet ou anxieux-se pendant la prise de sang ?

	Pas du tout	Un peu	Moyennement	Beaucoup	Extrêmement	Not Applicable

0 1 2 3 4 5 6 7 8 9 10

Anxiété hétérorapportée parent ()

42 Vous même, aujourd'hui, combien étiez-vous inquiet ou anxieux pendant la prise de sang? (Si 2 parents répondent, mettre le score le plus élevé)

	Pas du tout	Un peu	Moyennement	Beaucoup	Extrêmement	Not Applicable
	0	1	2 3	4 5 6	7 8	9 10
Anxiété du parent post ()						

40 Selon vous, combien votre enfant est-il inquiet ou anxieux habituellement pendant une prise de sang ou vaccin, lorsqu'il va chez le médecin?

	Pas du tout	Un peu	Moyennement	Beaucoup	Extrêmement	Not Applicable
	0	1	2 3	4 5 6	7 8	9 10
anxiété trait enfant hétérorapportée ()						

Page Break

41 Combien étiez-vous, vous-même, inquiet ou anxieux pendant vos vaccins (ou autres procédures médicales) en tant qu'enfant? (Si 2 parents répondent, mettre le score le plus élevé)

	Pas du tout	Un peu	Moyennement	Beaucoup	Extrêmement	Not Applicable
	0 1 2 3 4 5 6 7 8 9 10					
Anxiété trait du parent ()						

45 Combien êtes-vous satisfait de la prise en charge du bien-être de votre enfant (uniquement) pendant sa prise de sang?

	Pas du tout	Un peu	Moyennement	Beaucoup	Extrêmement	Not Applicable
	0 1 2 3 4 5 6 7 8 9 10					
Satisfaction parent ()						

End of Block: Questionnaire pour Parents (post)

Start of Block: Commentaires sur le patient

58 Commentaires supplémentaires:

____

End of Block: Commentaires sur le patient
